# Auditing Nicaragua’s anti-corruption struggle, 1998 to 2009

**DOI:** 10.1186/1472-6963-11-S2-S3

**Published:** 2011-12-21

**Authors:** Jorge Arosteguí, Carlos Hernandez, Harold Suazo, Alvaro Cárcamo, Rosa Maria Reyes, Neil Andersson, Robert J Ledogar

**Affiliations:** 1 CIETinternational in Nicaragua, Plaza la Palmera, Modulo 3, Managua, Nicaragua; 2 Centro de Investigación de Enfermedades Tropicales, Universidad Autónoma de Guerrero, Calle Pino, Colonia El Roble, Acapulco, Guerrero, Mexico; 3 CIETinternational, 511 Avenue of the Americas #132, New York, NY 10011, USA

## Abstract

**Background:**

Four social audits in 1998, 2003, 2006 and 2009 identified actions that Nicaragua could take to reduce corruption and public perception in primary health care and other key services.

**Methods:**

In a 71-cluster sample, weighted according to the 1995 census and stratified by geographic region and settlement type, we audited the same five public services: health centres and health posts, public primary schools, municipal government, transit police and the courts. Some 6,000 households answered questions about perception and personal experience of unofficial and involuntary payments, payments without obtaining receipts or to the wrong person, and payments "to facilitate" services in municipal offices or courts. Additional questions covered complaints about corruption and confidence in the country's anti-corruption struggle. Logistic regression analyses helped clarify local variations and explanatory variables. Feedback to participants and the services at both national and local levels followed each social audit.

**Results:**

Users' experience of corruption in health services, education and municipal government decreased. The wider population's perception of corruption in these sectors decreased also, but not as quickly. Progress among traffic police faltered between 2006 and 2009 and public perception of police corruption ticked upwards in parallel with drivers' experience. Users' experience of corruption in the courts worsened over the study period -- with the possible exception of Managua between 2006 and 2009 -- but public perception of judicial corruption, after peaking in 2003, declined from then on. Confidence in the anti-corruption struggle grew from 50% to 60% between 2003 and 2009. Never more than 8% of respondents registered complaints about corruption.

Factors associated with public perception of corruption were: personal experience of corruption, quality of the service itself, and the perception that municipal government takes community opinion into account and keeps people informed about how it uses public funds.

**Conclusions:**

Lowering citizens' perception of corruption in public services depends on reducing their experience of it, on improving service quality and access and -- perhaps most importantly -- on making citizens feel they are well-informed participants in the work of government.

## Background

We conducted four social audits on corruption in Nicaragua over an eleven-year period from 1998 to 2009. Companion articles explain the social audit concept [1] and report on other corruption-related social audits [2,3].

The Republic of Nicaragua is spatially the largest country in Central America, bordered by El Salvador and Honduras to the north, Costa Rica to the south, the Pacific Ocean to the west and the Caribbean Sea to the east. One quarter of its population of 6 million people lives in the capital region of Managua. The economy is primarily agricultural with beef and dairy products, coffee, seafood, beans, groundnuts and sugar as its primary export crops. Gold exports are another important source of revenue [4]. In 1998 Hurricane Mitch killed thousands, rendered 20% of the population homeless and caused billions of dollars worth of damage. Its 2009 gross domestic income (GDI) per capita according to the World Bank was US$1,070, the second lowest in the western hemisphere [5]. Nevertheless, Nicaragua ranks higher on the United Nations Development Programme's Human Development Index than its near neighbour, Guatemala, with a per capita GDI two and a half times as large [6].

We conducted four social audits under the auspices of somewhat different entities but always with governments of the time. The first audit took place during the presidency of Arnoldo Alemán (1997-2002), the second and third during the presidency of Enrique Bolaños (2002-2007) and the fourth during the second presidency of Daniel Ortega (2007 - present).

Transparency International (TI) has been a severe critic of corruption in Nicaragua [7]. Among the increasing number of countries included in TI's corruption index during the years from 1998 to 2010, Nicaragua's ranking for "perceived corruption" has remained essentially the same over that period. In 2010 its ranking improved slightly (127) compared to the previous year when it was given the same rank as Nigeria (130). TI's focus is on grand corruption, emphasising legislative and institutional weaknesses and large-scale bribery; its rankings are "perceived levels of corruption, as determined by expert assessments and opinion surveys" [8].

Three surveys conducted in 2004 and 2006 and 2008 by Vanderbilt University's Latin America Public Opinion Project (LAPOP), asked respondents if they were asked to pay bribes during the previous year and how widespread they considered corruption in the country. The proportion asked to pay bribes remained very similar over that period, varying between 16% and 18% [9,10].

We measured both perception and personal experience of corruption in five public services over an eleven year period using a different set of indicators. Instead of open requests for bribes we looked at unofficial and involuntary payments, payments made without obtaining receipts or to the wrong person, and payments to "facilitate" a service in a municipal office or a court.

Our four social audits studied a sample of 6,000 households in 71 clusters representative of the country's seven geographical regions in 1998, 2003, 2006 and 2009. From the beginning, these surveys sought actions that could be taken by government authorities to decrease both corruption itself and public perception of it. We audited the same five public services in all four cycles: health centres and health posts, municipal government, public primary schools, transit police and the courts.

## Methods

### The sample

Using the 1995 census as the sample frame for the seven geographical regions (West, Pacific-south, Segovias, North, Centre, Managua and Caribbean coast), we stratified by type of settlement (regional capital, departmental centre, municipality centre, rural settlement) proportional to population. The last stage of selection was purposive: a regional committee with knowledge and experience selected panels of sites they agreed to represent the spread of physical and socio-economic conditions in each stratum. We used the same geographical sample in each of four measurement cycles. There was no sampling within the clusters; we surveyed all households in each cluster.

The original 70 sites became 71 when we divided a neighbourhood in Matagalpa into two sites. There was no change in the settlements covered by each of the four surveys. For analysis, we weighted each site in proportion to the sample and to the population.

### The survey

We designed the survey instruments (household questionnaire and key informant interview guide) in consultation with technicians from government and civil society.

Questions covered both experience and perception of corruption in the five services; health centres and health posts, education, municipal government, transit police and the courts. (In 2009, at the request of authorities, we included three new government services in the survey: the Office of the Comptroller General, the Public Prosecutor's Office and the *Procuraduría General de la República* which represents the state in all business negotiations. We also added some questions widening the scope of the inquiry into primary education and the police force. This article reports only on answers to questions raised in more than one of the four surveys.)

Six survey teams composed of three interviewers, a supervisor and a support worker conducted the household surveys, capturing responses in Bhopal books [11,12]. We determined the number of households to survey in each type of settlement by the number that a team travelling in one vehicle could cover in one day. In rural communities this resulted in 60 to 70 contiguous households. In the municipal and departmental centres the number was 100 to 110 and in Managua it averaged 130.

Training of field workers focused on the interview process, recording responses in the Bhopal book and on ethical issues that might arise from the interview including confidentiality and respect. As far as possible, the same interviewers returned to the same settlements in each of the four surveys. Refresher training before each of the post-1998 surveys updated the skills of the veterans and incorporated any new interviewers into the process. Training went hand in hand with piloting in neighbourhoods outside those of the sample. Supervisors had to certify that newcomers were fully trained before field work could begin.

### Data entry and analysis

Data entry relied on Epi-Info, the public domain data entry and analysis package. Double data entry and verification of discordant records minimised key-stroke errors. Further cleaning looked for logical inconsistencies and out-of-range responses. Analysis relied on Epi-Info in the first cycle and subsequently on CIETmap, open source software that combines epidemiological analysis with raster and vector mapping [13]. Initial analysis generated weighted frequencies of the main indicators about service use and perceptions and experience of corruption.

We reported contrasts as adjusted odds ratio (ORa); 95% confidence intervals (CI) were those of Cornfield; and interaction was assessed using the test of Woolf [14]. We tested the significance of differences in levels of indicators from one year to another using the Mantel-Haenszel Chi square (X^2mh^) procedure [15]. We calculated gains as the product of the risk difference (RD) and the proportion requiring intervention (PRI). We did not adjust for clustering.

Conditioning variables were: sex, age and educational level of the person interviewed; geographic region; degree of urbanization; poverty levels; user/non-user of services during the time period; participation in any organisations; perceiving that municipal authorities take community opinions into account; and feeling informed - and by what medium - about use of municipal funds.

### Feedback

**Figure 1:**
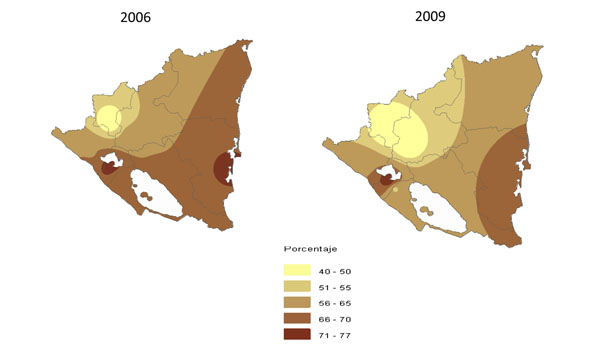
Geographic change from 2006 to 2009 in respondents' perception of corruption in any of the Nicaraguan public services investigated.

Interviewers returned to the same households they had interviewed to distribute a pamphlet summarising the aggregate results of each cycle. We presented results at the national level to the authorities responsible for oversight of municipal government, the health and education ministries, the national police and the court system. We discussed results specific to each geographical region at regional workshops with representatives of each regional department and each of the services that were the subject of the audit. We used population-weighted raster maps as appropriate during these presentations. Figure 1 gives an example of these maps.

### Ethical considerations

Social audits only deal anonymously with occurrence of corrupt practices. They do not name perpetrators. To avoid reprisals we do not report data at the cluster level. The lowest level of disaggregation at which we reported data from these surveys was by geographic region or settlement type. At the time these social audits begun, Nicaragua had no mechanism for review of this kind of research. Therefore, the ethics committee of the *Centro de Investigación de Enfermedades Tropicales* (CIET) of the *Universidad Autónoma de Guerrero* in Mexico gave ethical clearance for these surveys.

## Results

### Health centres and health posts

The proportion who received attention at public health centres and posts in the six months before the survey fluctuated from 59% in 1998 to 63% in 2003 and down to 54% in 2006; but then it increased substantially in 2009 to 75%.

The proportion who reported being asked to make a financial contribution for services received at a health centre or health post declined steadily over the eleven-year period. In 2009 it was only 2%.

**Figure 2:**
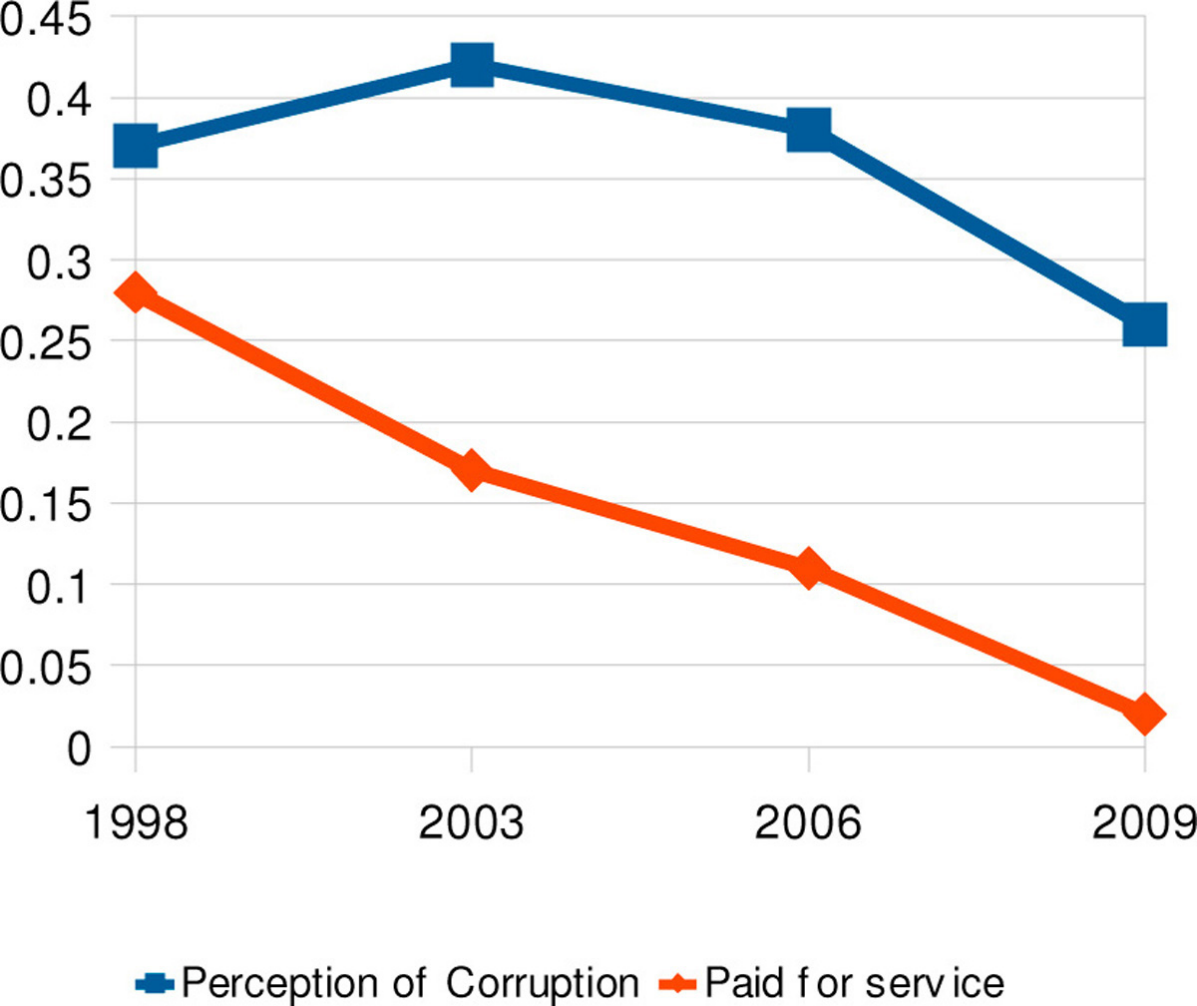
Perception of corruption in health centres compared with users' having paid for the service, 1998-2009

It took some time, however, for perception of corruption in the health services to decline. It increased between 1998 and 2003 while experience of corruption was declining quite steeply. Only after that did trend in perceptions begin to match that of users' experience more consistently (Figure 2).

In 1998, 2003 and 2006 we asked respondents if anyone in these health services had asked them for a contribution and, if the answer was affirmative, whether they were given a receipt for the contribution. In light of a new policy of totally free health services established in 2007, the 2009 survey simply asked the first of these questions.

In 2006, respondents asked to make a financial contribution to a health centre or health post were 60% more likely to perceive corruption in these services (ORa 1.6, 95% CI: 1.2-2.0). In 2009 perception of corruption in health centres and health posts was associated with the perception that community opinions were not taken into account by municipal governments (ORa 1.7, 95% CI: 1.4-2.0).

Satisfaction with the service received at health centres and health posts was also associated with perception of corruption in those services (2006 ORa 1.8, 95% CI 1.4-2.4; 2009 ORa 2.9, 95% CI 2.6-3.3).

### Public primary schools

The proportion of households with at least one child between the ages of 7 and 12 matriculated in a public primary school remained around 70-80% over the eleven-year period.

We asked about perception of corruption in the schools in all four cycles. In 1998, 2003 and 2006 we asked participants if they made any monetary contribution to the school in the past year and if so whether they contributed out of their own choice or if they were obliged to do so. In light of a 2007 presidential decree that all primary and secondary education was free, our 2009 questionnaire simply asked whether respondents paid anything and we considered any payment made to have been involuntary.

**Figure 3:**
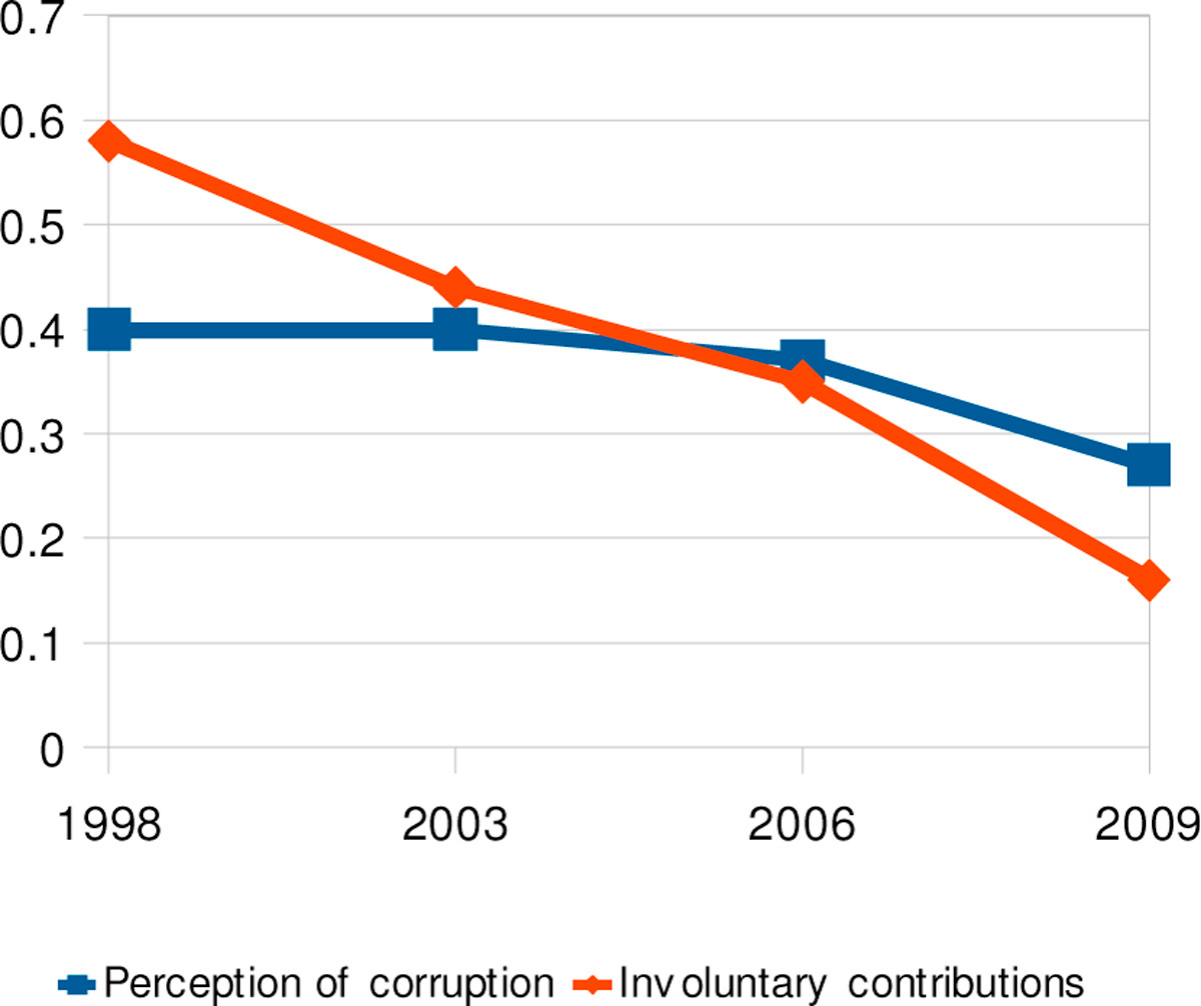
Perception of corruption in public primary education compared with parents' involuntary contributions to school expenses, 1998-2009

The population's perception of corruption in public primary schools stayed constant between 1998 and 2003, decreased somewhat from 2003 to 2006 and dropped more steeply after that. Experience of corruption, in the form of involuntary contributions to school expenses, decreased steadily from the first survey cycle in 1998 (Figure 3).

Perception of corruption in primary schools was associated with being asked for an involuntary monetary contribution (2003: ORa 1.8, 95% CI 1.5-2.1;2006: ORa 1.4, 95% CI 1.0-1.9; 2009: ORa 2.7, 95% CI 2.2-3.5).

Around 60% of respondents expressed satisfaction with quality of education over the eleven-year period of our surveys. Perception of teaching quality and perception of corruption were closely associated. In 2006, a respondent who considered that the teaching quality was "bad" was 14 times as likely to consider the educational system corrupt compared with one who thought the teaching was good (ORa 14.1, 95% CI 4.4-45.4).

### Municipal government

Close to 35% of responding households in all four social audits conducted some type of business with municipal government during the 6 month before the survey.

**Figure 4:**
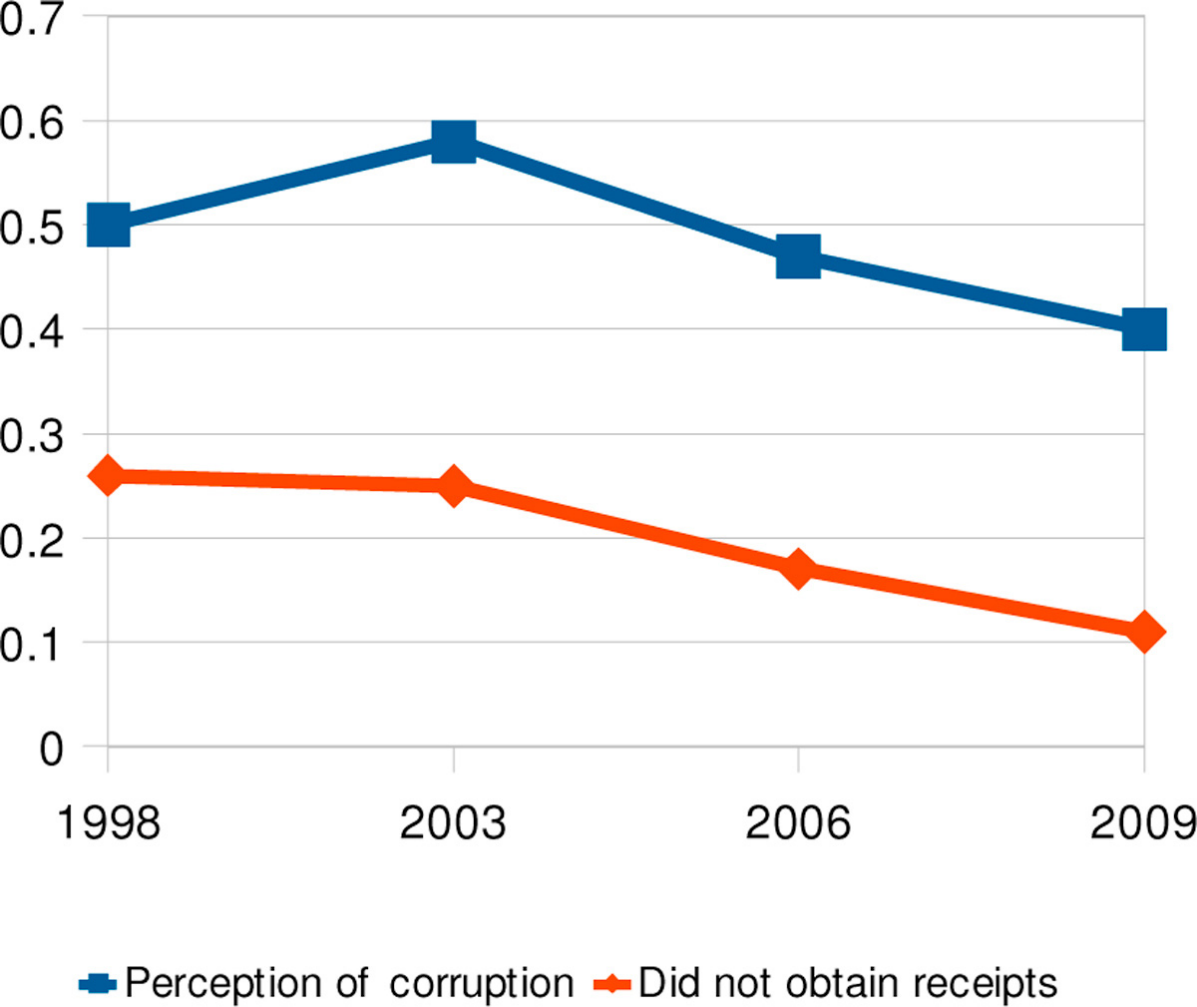
Perception of corruption in municipal government compared with users' getting receipts for payments made, 1998-2009

Public perception of corruption in municipal government increased between 1998 and 2003 but diminished consistently from then on (Figure 4). Perception of corruption in municipal government was highest among residents of the Managua region.

We asked respondents whether they paid anything to facilitate whatever business they had with the municipalities and, if so, whether they received a receipt for it. The proportion who paid for facilitating the process increased from 19% to 33% from 1998 to 2003 and after that remained at roughly this one-third level. On the other hand, the proportion that did not receive receipts for their payments decreased, only slightly from 1998 to 2003 but then more markedly afterwards (Figure 4). In 2009 a user who did not receive a receipt for a payment was 80% more likely to consider municipal government corrupt compared with one who did receive a receipt (ORa 1.8, 95% CI 1.1-3.2).

The proportion of respondents who felt that municipal governments took community opinions into account doubled from 18% to 37% between 2003 and 2006 and rose slightly higher in 2009. In 2003, 2006 and 2009, this perception was associated with not perceiving corruption in any of the services under consideration as well as by overall confidence in the government's anti-corruption struggle (Table 1).

**Table 1:** Perception that municipal governments take community opinion into account: associations with perceptions that services were *not *corrupt and with confidence in the anti-corruption struggle (all respondents 2003, 2006, 2009)

	2003		2006		2009	
	**ORa**	**95%CI**	**ORa**	**95%CI**	**ORa**	**95%CI**

Perception of no corruption in public services*	2.4	1.9-3.1	2.6	2.2-3.1	1.8	1.5-2.2
**Confidence in the anti-corruption struggle**	1.7	1.5-2.0	1.6	1.2-2.1	1.8**	1.6-2.0

* Respondents who did not perceive corruption in any of the services under investigation by this social audit.

** The association was stronger (OR 2.7) among those who felt informed about how municipal funds were being used.

Regarding municipal government alone, the feeling in 2009 that municipal governments took one's opinions into account was strongly associated with *not* considering municipal government corrupt (ORa 3.0, 95% CI 2.7-3.4). Perceiving that municipal government took into account community opinions was, in turn, associated with perception of being informed about use of municipal funds. A respondent who felt informed on the use of funds was five times as likely to feel that his/her opinions were taken into account compared with one not informed (ORa 5.4, 95% CI; 531/718 informed vs. 1848/5332 uninformed respondents who feel their opinions taken into account). If this size of difference could be reproduced in a large randomised controlled trial, this would imply that if all citizens were to feel informed about the use of public funds by municipalities, the theoretical gain would be a third more of them who perceive that local government was taking their opinions into account (RD 0.393, PRI 0.8813, Gain 0.346).

**Figure 5:**
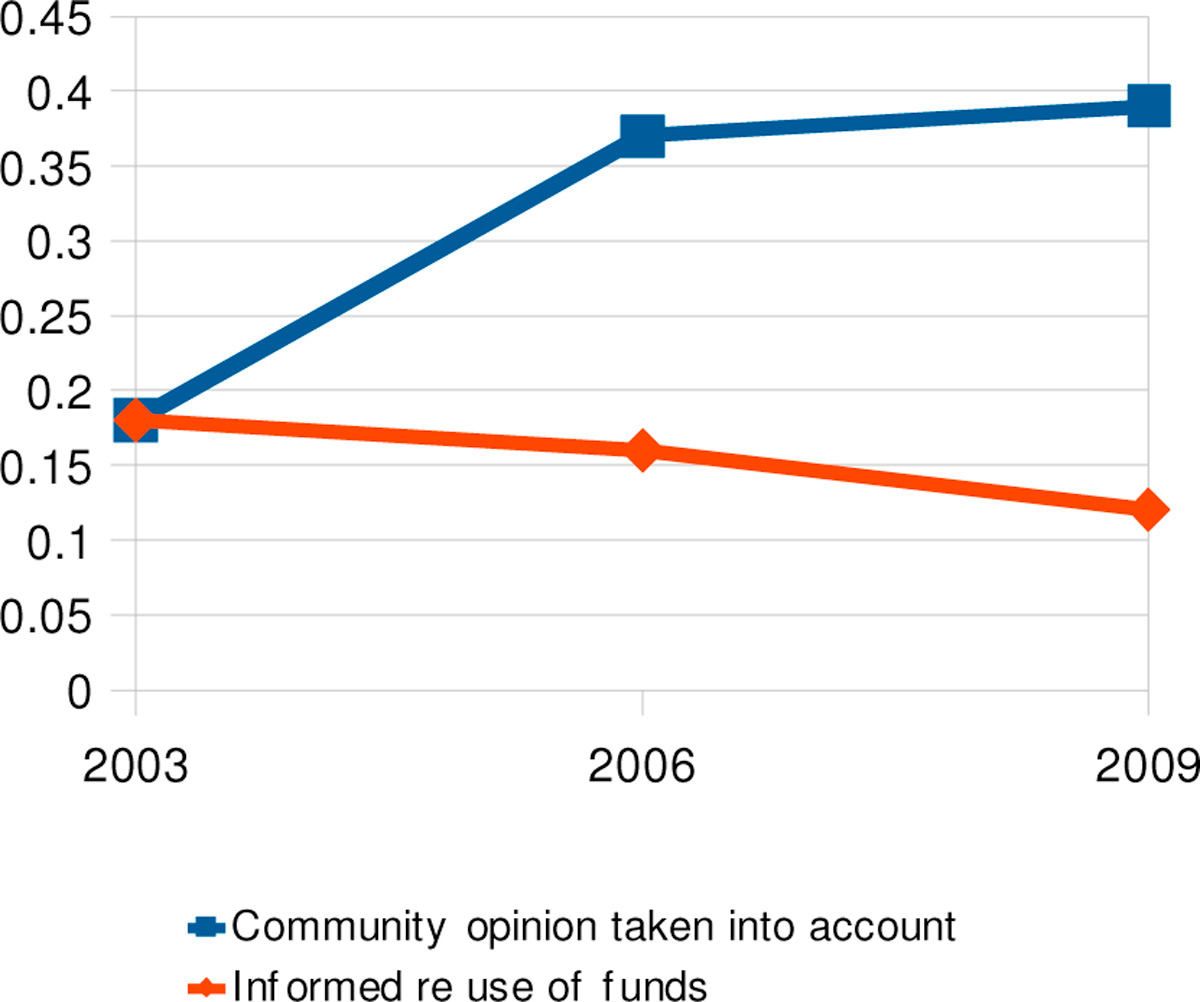
Contrasting trends in perceptions that community opinions were taken into account and feeling informed about use of municipal funds, 2003-2009.

At the same time, the proportion of households reporting that they felt informed about how municipal governments were using public funds had declined from 18% to 16% to 12% between 2003 and 2009. Figure 5 juxtaposes the trend exhibited by these two related indicators from 2003 to 2009.

### Police

**Figure 6:**
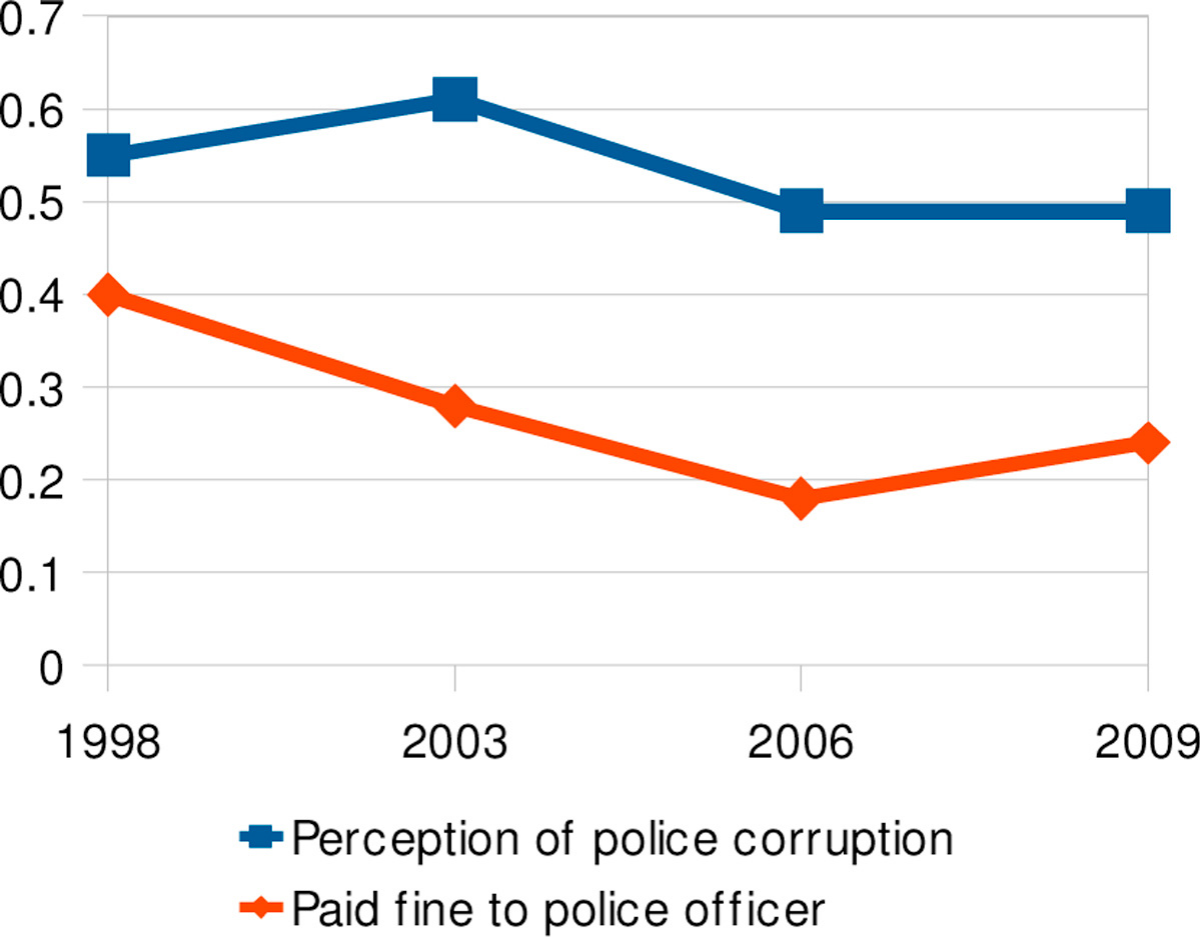
Perception of corruption in police force compared with drivers having paid fines to police officers directly for traffic violations, 1998-2009

Among households where someone had a driver's licence, the proportion with someone stopped and fined for a traffic infringement during the previous six months was 24%, 16%, 19% and 25% respectively in 1998, 2003, 2006 and 2009. The proportion reporting they made direct payments to transit police officials declined from 1998 to 2006 but then rose between 2006 and 2009. The perception of corruption among all respondents increased from 1998 to 2003 while drivers' experience of it was declining, but thereafter the perception tracked drivers' experiences more closely (Figure 6).

Factors associated with perception of corruption in the police force included having had to make direct payments to police officers (2003 ORa: 1.9, 95% CI 1.0-3.2; 2009 ORa 1.9, 95% CI 1.3-3.0), having learned of corrupt behaviour by high-level public officials in the previous month (2006 ORa 2.5, 95% CI 2.2-2.9) and absence of rural judicial facilitators (ORa 1.3, 95% CI 1.1-1.7).

### Courts

The proportion of households where a member had business with the court system during the two years before the survey stayed between 14% and 18% over the eleven year period. We asked respondents whether they had to pay to facilitate the court procedure. Among those who said yes in 2009, three-quarters paid through lawyers and 20% directly to a court official.

**Figure 7:**
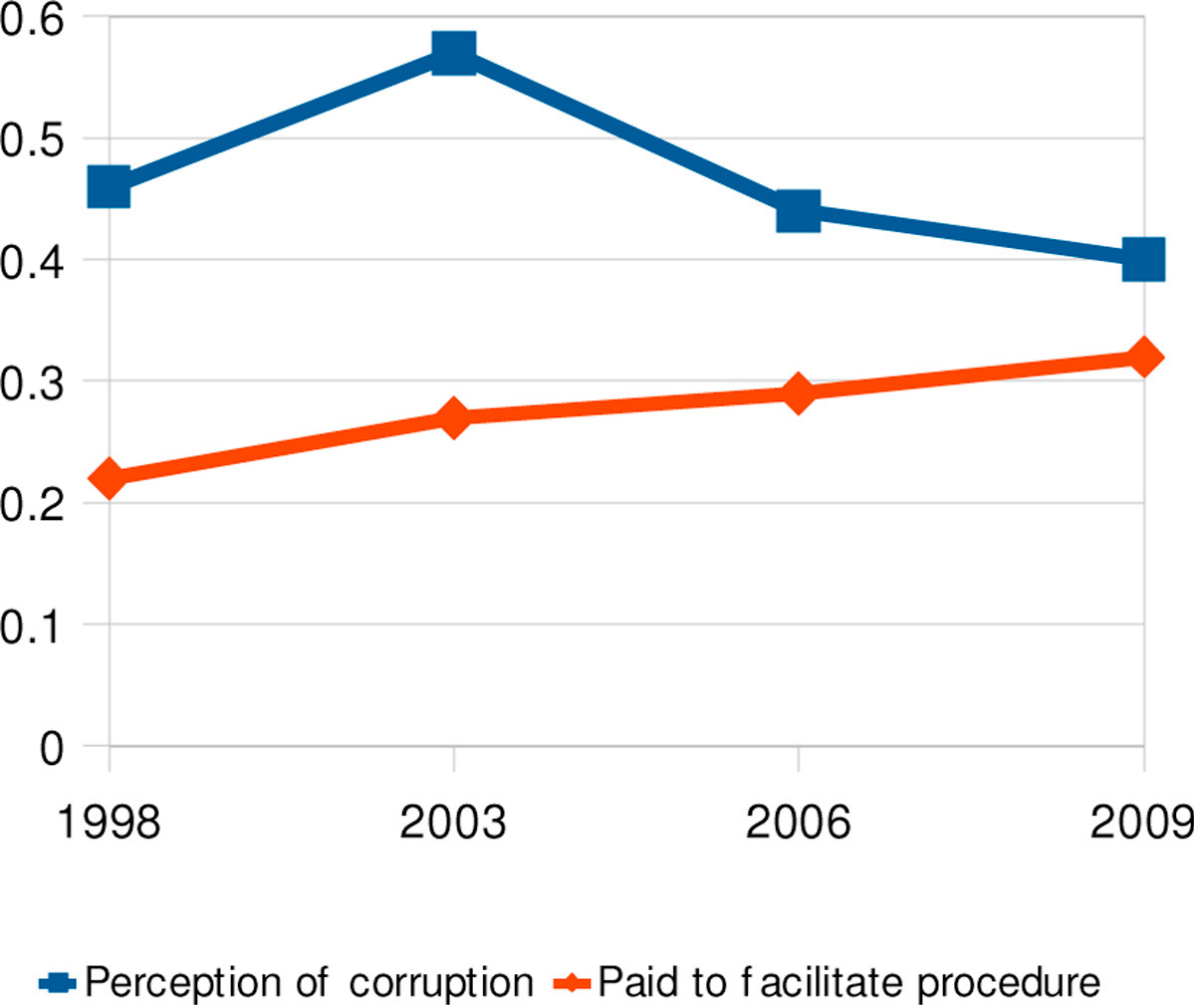
Perception of corruption in the court system compared with users having paid to facilitate a procedure, 1998-2009.

Perception among all respondents of corruption in the courts increased from 1998 to 2003 but declined thereafter despite a modest but steady overall increase in cases where users of the courts had to make payments to facilitate their procedures (Figure 7).

In Managua between 2006 and 2009 there was an 8% decrease in the proportion of users who had to pay to facilitate a procedure, but because of the small numbers involved we cannot exclude the possibility that this was a chance effect. Managua, where the perception of corruption in the judiciary had previously been higher than anywhere else in the country, also experienced the steepest decline in that perception from 2006 to 2009 (2006: 796/1113; 2009: 699/1113; Χ^2mh^ 19.2).

There was an association between users who paid and the broader perception of corruption. In 2006 and 2009 a respondent who paid to facilitate a procedure was roughly twice as likely to perceive corruption in the court system as one who had not paid (2006 ORa 1.8, 95% CI 1.2-2.8; 2009 ORa 2.0, 95% CI 1.4-2.7).

Other factors that influenced perception of corruption in the courts in 2006 were: having heard about corruption among high-level public officials during the preceding month (ORa 2.8, 95% CI 2.3-3.4) and absence of judicial facilitators (ORa 1.3, 95% CI 1.0-1-6).

### Confidence in the anti-corruption struggle

Beginning in 2003, we asked respondents if they had confidence in the anti-corruption struggle. Confidence in the struggle increased steadily, if not dramatically, from 5 to 6 out of 10 between 2003 and 2009. Respondents older than 20 years of age and better educated respondents were less likely to express confidence in the struggle (X^2mh^ 8.9 and 15.9 respectively).

Factors associated with lack of confidence in the anti-corruption struggle in 2009 included:

• having been affected by an act of corruption in some government institution (ORa 2.2, 95% CI 1.8-2.7)

• having made an unauthorised payment to any of the public services under consideration in this study (ORa 1.3, 95% CI 1.1-1.5)

• perceiving that educational materials in the primary schools (ORa 1.5, 95% CI 1.3-1.7) or medicines in health centres and health posts (ORa 1.6, 95% CI 1.4-1.8) were not handled "honourably".

### Complaints about corruption

We asked respondents whether they had ever registered a complaint about any act of corruption and, if not, why not. Affirmative answers were 5% in 1998, 8% in 2003, 7% in 2006 and 5% in 2009. Among those who did register a complaint in 2009 only 36% expressed satisfaction with the outcome. Reasons given for not registering a complaint included: lack of a reason to do so, indifference, distrust of the system, fear of reprisal, ignorance of the proper procedure, logistic difficulties and lack of proof.

## Discussion

### Study limitations

The sample was highly stratified but last stage sample selection was purposive, based on a regional committee consensus. A sampling bias cannot be ruled out although this would be consistent in each of the four measurement years and should not have affected the time-trends or associations reported here.

We made no adjustments for a possible clustering effect during analysis.

### Overall progress in the struggle against corruption

The study shows a fairly steady decrease in users' experience of corruption in health services, municipal government and education. The wider population's perception of corruption in these sectors decreased as well, but not as quickly as did experience itself. One possible explanation of this phenomenon is that, in addition to their own experience, people may need repeated confirmation from others that the practice of corruption has diminished before their opinions about corruption are likely to change. Another is the effect of local political discourse and communication media on public opinion. Progress being made by the traffic police reversed between 2006 and 2009 and perception of corruption among the police ticked upwards in parallel to drivers' experience. Experience of corruption in the courts got worse over the eleven-year period of this study, with the possible exception of Managua between 2006 and 2009. Respondents' perception of judicial corruption, after peaking in 2003, declined from then on. Confidence in the country's anti-corruption struggle grew from 50% to 60% between 2003 and 2009. The proportion of respondents who registered complaints about corruption never rose higher than 8% over the entire period.

### Health centres and health posts

Health care in Nicaragua was decentralised in the 1990s with the encouragement and support of the World Bank and the Inter-American Development Bank. Decisions about fees charged were made at facility level [16], with sliding scales for user contributions according to the client's socio-economic status [17]. The complexities of such a system may help to explain why perception of corruption increased between 1998 and 2003 while the proportion asked for a financial contribution without issuance of a receipt decreased significantly over the same period. This decline continued between 2003 and 2006 but the latter period also saw a decline in the proportion of respondents actually using the health centres and health posts.

The last three years covered by our survey saw a marked increase in use of services and the near disappearance of user charges. These years coincide with the new health policy explicitly to increase access to primary health care services, conceived as a basic right of all citizens [18]. The 2% involuntary payment figure reported for 2009 is the proportion of respondents who made any payment at all.

### Education

Under the same influence as affected primary health services, Nicaragua decentralised its educational system during the decade of the 1990s. The new system gave a certain degree of control to local school councils with parental participation, but it also gave these councils responsibility for raising substantial portions of the schools' operating budgets. The 2002 law that codified the decentralisation declared that all Nicaraguans should have access to free primary education but at the same time, called the schools "community participation schools", thus legitimising some voluntary monetary contributions from parents [19]. For this reason our 1998, 2003 and 2006 surveys distinguished between voluntary and involuntary contributions; we considered only the involuntary ones as indicators of corruption. After the declaration that basic education was totally free in 2007, our 2009 survey considered any payment to be involuntary. In 2009 involuntary contributions were still strongly associated with people's perception of corruption in the primary education system.

### Municipal government

Progress in both perception and experience of corruption in municipal government occurred against a background of significant legal and fiscal changes during the period of this study. Municipalities were given some direct responsibilities for their own management for the first time as a result of the 1995 Law of Municipal Autonomy. The first municipal elections occurred in 1996 but it was only beginning in 2003 that central government allocate any substantial amounts of their revenue to enable municipalities to fulfil their mandates [20]. Public perception that municipal governments were taking community opinions into account increased between 2003 and 2006. This coincided with an increase in experience of obtaining receipts for payments and with a decrease in perception of corruption in municipal government.

The association between the perception that municipal governments take community opinions into account and perception of municipal corruption is one of the key actionable findings of these surveys. Table 1 suggests that public perception of being listened to by municipal officials might help to decrease the perception of corruption not only in municipal government but also in other government services. Furthermore, policies of informing people about how municipalities use public funds can influence perception of being taken into account and, in turn, the overall perception of corruption not just in municipal government but in other public services as well.

### Traffic police

The general perception of corruption in the police force, which was on the decline between 2003 and 2006, levelled off from 2006-2009 while an increasing proportion of drivers made direct payments to the traffic police.

Drivers' experiences with the traffic police is only one point of encounter between the public and the police force. Starting in 2009 we introduced three new questions on the police: households that had any dealing with the police force in the previous year and the nature of that encounter, opinions on the work of the police, and payments for facilitating any service requested of the police. But these are not discussed here because there is as yet no point of comparison.

### The courts

Despite a steady, if modest, rise in the proportion of users who paid something to facilitate a court procedure (and the association between having made a payment to facilitate and the perception of corruption), the overall perception of corruption in the judiciary, after rising from 1998 to 2003, decreased markedly between 2003 and 2006 and continued to do so at a lesser rate in the following three years.

A partial explanation for this paradox could be that a very popular programme of "rural justice facilitators", promoted by the Organization of American States [21]- started in 1998 with the express purpose of improving access to justice for all and speeding up the judicial system by reducing the backlog of cases. Since the programme expanded to all parts of the country only gradually, the downward shift in perception of judicial corruption from 2003 roughly coincided with the period of its roll-out [22,23]. In 2006 communities that did not have judicial facilitators were more likely to perceive corruption in the judiciary.

In the specific case of Managua, the decrease in unofficial payments and perception of corruption in the courts registered between 2006 and 2009 may be related to the introduction in June 2007, with support from the Inter-American Development Bank, of an new model for management of judicial procedures in the Managua courts [24].

### Confidence in the anti-corruption struggle

This indicator is not tied to any particular government service discussed in the questionnaire. It refers in principle to every aspect of the country's effort to combat corruption. The way people responded to the question was associated with both recent awareness of corrupt behaviour on the part of high-level public officials and individual experience of corruption in one or other of the services under consideration. The increase of confidence in the anti-corruption struggle from 2003 to 2009 manifested itself in all settlement types and in all geographic regions.

A different impression is given by the LAPOP surveys that depict a perception that corruption was becoming more widespread in Nicaragua from 2004 to 2008. The LAPOP surveys asked: "Keeping in mind your personal experience or experiences you have heard about, is corruption among public officials: (1) Very widespread (2) Somewhat widespread (3) Not very widespread (4) Not at all widespread?" The proportions of LAPOP respondents who considered corruption very widespread were 49.1% in 2004, 66.1% in 2006 and 74.3% in 2008.

We did not ask about the *extent* of corruption among public officials. We asked whether respondents had confidence in the country's anti-corruption efforts. The two questions are not the same but our results do tend to give a more positive picture of corruption in Nicaragua than those of LAPOP, perhaps because they were asked in the context of a search for actionable measures at national and local levels to carry the struggle against corruption even further.

### Complaints

We were unable to detect any progress over the eleven-year period in people's taking the initiative to lodge complaints about corruption. Government could do a great deal more to encourage and make it easier for citizens to register complaints. Specifically, it could establish complaint mechanisms and inform citizens about how to use them anonymously and securely. For this to be effective, government must also act upon complaints once they have been registered and verified and it must inform the complainant of the result. Complaints unheeded (or worse) feed cynicism and indifference.

### Social Audits and the struggle against corruption

The CIET social audits have been only one of several instruments in the Nicaraguan struggle against corruption. At certain moments, however, the audits have played a perceptible role in that struggle:

✯ With input from the 1998 baseline social audit, the then National Integrity Committee convened a National Integrity Forum from which emerged a National Integrity Plan, approved in May 1999. The Plan resulted in a series of laws including the State Contracts Law which established a system for tendering government contracts for public bidding [25], and the law governing the organization, competence and procedures of Nicaragua's executive branch [26].

✯ The data from the 2006 survey constituted one of the lobbying tools used by advocates for what became the Access to Public Information law of 2007 [27].

✯ This feedback process continued in 2009 with presentations of the evidence to the relevant institutions and training centres such as the Police Academy. This evidence fed into measures such as the Code of Ethics for Public Servants, proclaimed by Executive Decree 35-2009 [28].

Particular pieces of evidence from the social audits appear to have been influential at key moments:

✯ Evidence of how the public perceived the "contributions" they were asked to make for health and education services as "involuntary payments" probably gave some impetus to the move toward eliminating all fees for primary education and basic health services.

✯ Feedback to authorities from the 2003 survey onward emphasised the differences among regions where progress had occurred compared to others where there was no progress. Changes in perception of corruption at the local level such as those depicted in Figure 1 appear to reflect the effects of such regional feedback.

In addition:

✯ CIET's report on the 2009 social audit was made available to the Nicaraguan public on the website of the *Procuraduría General de la República* which represents the state in all business negotiations [29].

✯ At the time we were completing the final draft of this article negotiations were under way for a fifth round of social audit on corruption.

## Conclusions

Governments need to deal with both the experience and perception of corruption. Reducing citizens' experience of corruption is the more essential task and there are practical tools for doing so, such as clear and accessible information about use of public funds and prices for services, extending receipts for all payments, and reducing and clarifying the role of "facilitators" in public transactions. Lowering citizens' perception of corruption in public services depends most of all on reducing their experience of it, but also on improving the quality of services and access to services.

The degree to which citizens feel that they are participants in the work of government also influences their perception of corruption. Clear and adequate information about how government uses public funds enhances that sense of participation. A lack of transparency on how authorities use the funds entrusted to them can undermine efforts to build up the public's sense that their government takes community opinions into account. Thus the decline from 2003 to 2009 in respondents' sense of being informed about the use of municipal funds is a threat to Nicaraguan citizens' sense of participation in local government.

Central government's capacity to take the needed remedial measures is very limited. Reforms need to be implemented at regional, district, municipal and even neighbourhood levels and the results monitored periodically. Repeated social audits of the kind described here are a useful tool for use by governments who are serious about dealing with corruption in their midst.

## Competing interests

The authors declare they have no competing interests.

## Authors' contributions

JA managed all four audits and the analysis of their results. CH participated in the design and analysis of results of the 2006 and 2009 audits. HS was field coordinator of the 2006 and 2009 audits, helped to analyse their data and helped edit their final reports. AC participated in all four audits, coordinating data entry, supervising field teams and contributing to survey design. RMR took part in all four audits in various roles including field supervision and contributions to survey design. NA was responsible for the overall design and technical oversight of all four studies. RJL was responsible for administrative supervision of the studies, did background research and wrote this report on the basis of the field team’s Spanish-language reports from the four studies. All authors reviewed and agreed with the final draft.

## List of abbreviations used

CI: Confidence intervals; GDI: Gross domestic income; LAPOP: Latin America Public Opinion Project; ORa: Adjusted odds ratio; PRI: Proportion requiring intervention; RD: Risk difference; TI: Transparency International; X^2mh^: Mantel-Haenszel chi square

## References

[B1] Andersson N Building the community voice into planning: 25 years of methods development in social audit BMC Health Services Research 2011 11 Suppl 2 S1 22376121 10.1186/1472-6963-11-S2-S1 PMC3397387

[B2] Paredes-Solís S Andersson N Ledogar RJ Cockcroft A Use of social audits to examine unofficial payments in government health services: experience in South Asia, Africa, and Europe BMC Health Services Research 2011 11 Suppl 2 S12 22376233 10.1186/1472-6963-11-S2-S12 PMC3332556

[B3] Paredes-Solís S Villegas-Arrizón A Ledogar RJ Delabra-Jardón V Álvarez-Chávez J Legorreta-Soberanís J Nava-Aguilera E Cockcroft A Andersson N Reducing corruption in a Mexican medical school: impact assessment across two cross-sectional surveys BMC Health Services Research 2011 11 Suppl 2 S13 22376281 10.1186/1472-6963-11-S2-S13 PMC3332557

[B4] Gobierno de Nicaragua Centro de Tramites de las Exportaciones (CETREX) http://web.worldbank.org/WBSITE/EXTERNAL/COUNTRIES/LACEXT/NICARAGUAEXTN/0,,contentMDK:22255024~pagePK:1497618~piPK:217854~theSitePK:258689,00.html

[B5] World Bank, Nicaragua Country Brief http://data.worldbank.org/WBSITE/EXTERNAL/COUNTRIES/LACEXT

[B6] United Nations Development Programme Human Development Report. 2010 http://hdrstats.undp.org/en/countries/profiles/NIC.html

[B7] Transparency International Los sistemas nacionales de integridad: Estudio de país - Nicaragua Transparency International 2008

[B8] Transparency International Corruption perception index. 2010. http://www.transparency.org/policy_research/surveys_indices/cpi/2010/results

[B9] Ortega M Hegg Castillo M Venerio Seligson MA The Political Culture of Democracy in Nicaragua, 2006. Universidad Centroamericana and LAPOP; 2007. http://www.vanderbilt.edu/lapop/ab2006/nicaragua1-en.pdf

[B10] Perez OJ Seligson MA Political Culture of Democracy in Nicaragua, 2008: The Impact of Governance. LAPOP, Inter-American Bank and UNDP; 2008. http://www.vanderbilt.edu/lapop/nicaragua/2008-politicalculture.pdf

[B11] Andersson N Ajwani MK Mahashabde S Tiwari MK Muir MK Mehra V Ashiru K Mackenzie CD Delayed eye and other consequences from exposure to methyl isocyanate: 93% follow up of exposed and unexposed cohorts in Bhopal British Journal of Industrial Medicine 1990 47 8 553 558 2393636 10.1136/oem.47.8.553 PMC1035230

[B12] Andersson N Kerr M Muir Mehra V Bhopal eye Lancet 1984 ii 1481 6151098 10.1016/s0140-6736(84)91685-4

[B13] Andersson N Mitchell S Epidemiological geomatics in evaluation of mine risk education in Afghanistan: introducing population weighted raster maps International Journal of Health Geographics 2006 5 1 10.1186/1476-072X-5-1 16390549 PMC1352365

[B14] Woolf B On estimating the relation between blood group and disease Ann Hum Genet 1955 19 251 253 10.1111/j.1469-1809.1955.tb01348.x 14388528

[B15] Mantel N Haenszel W Statistical aspects of the analysis of data from retrospective studies of disease J Natl Cancer Inst 1959 22 719 48 13655060

[B16] Bossert T Bowser D Corea L Studies of decentralization of the health system in Nicaragua: Final report USAID and Harvard School of Public Health 2001

[B17] Jack W Contracting for health services: an evaluation of recent reforms in Nicaragua Health Policy and Planning 2003 18 2 195 204 10.1093/heapol/czg024 12740324

[B18] Gobierno de Nicaragua Ministerio de Salud Política nacional de Salud: Política nacional de Salud. MINSA 2008. http://www.tortillaconsal.com/health_nicaragua.html

[B19] St Louis MJ Does education decentralization reform in Nicaragua influence household schooling decisions? Masters thesis Georgetown University, Washington DC; 2006. http://aladinrc.wrlc.org/bitstream/1961/3645/1/etd_mjs79.pdf

[B20] Roque JR Transferencias municipales: “Motor eficiente o deficiente”. El Observador Económico 2007. http://www.elobservadoreconomico.com/imprimir/304

[B21] Organización de Estados Americanos Programa Intgeramericano de Facilidadores Judiciales http://www.oeapifj.org/nicaragua.html

[B22] Westerlund S Widenbladh M The Rural Judicial Facilitators Programme in Nicaragua -- an exemplary model of restorative justice? UMEA University, Department of Law, MFS-report no 43 2007. http://www.jus.umu.se/digitalAssets/13/13607_sara-westerlund-marlene-widenbladh.pdf

[B23] Galo F De los facilitadores judiciales. La Prensa Managua; 2010. http://www.laprensa.com.ni/2010/06/08/opinion/27031

[B24] República de Nicaragua Poder Judicial Nuevo modelo de tramitación judicial en Managua http://www.poderjudicial.gob.ni/djudiciales/modelogestion.htm

[B25] República de Nicaragua Ley de Contrataciones del Estado http://www.cesdepu.com/foro/nicaragua.htm

[B26] República de Nicaragua Ley de Organización, Competencia y Procedimientos del Poder Ejecutivo http://www.dga.gob.ni/ley/Ley%20No%20290.pdf

[B27] República de Nicaragua Ley de Acceso a la Información Pública http://legislacion.asamblea.gob.ni/Normaweb.nsf/%28$All%29/675A94FF2EBFEE9106257331007476F2?OpenDocument

[B28] República de Nicaragua Decreto ejecutivo 35-2009 http://legislacion.asamblea.gob.ni/normaweb.nsf/9e314815a08d4a6206257265005d21f9/1d93cd4eaf137dac0625765c006f6a87?OpenDocument

[B29] República de Nicaragua Procuraduría General de República Percepción de Corrupción http://www.pgr.gob.ni/images/stories/2009/HALLAZGOS.pdf

